# Development of Biocompatible Fatty Acid-Based Ionic Liquids for the Effective Topical Treatment of Periodontitis

**DOI:** 10.1021/acsomega.5c09114

**Published:** 2025-12-29

**Authors:** Mayuko Yanagawa, Mayuka Nakajima, Mayumi Ikeda-Imafuku, Tatsuya Fukuta, Kotone Yoshimura, Chunyang Yan, Lorena Caceres Zegarra, Honoka Takikawa, Truong T. Thien, Ruka Koizumi, Koichi Tabeta

**Affiliations:** † Division of Periodontology, Faculty of Dentistry & Graduate School of Medical and Dental Sciences, 13145 Niigata University, Niigata 951-8514, Japan; ‡ Department of Physical Pharmaceutics, School of Pharmaceutical Sciences, Wakayama Medical University, Wakayama 640-8156, Japan

## Abstract

Periodontitis is a widespread chronic inflammatory disease characterized by the progressive destruction of tooth-supporting structures, ultimately causing tooth loss and impaired quality of life. Pathogenic microorganisms residing in the periodontal pockets are involved in disease progression. While antibiotics are widely used, the global rise of antimicrobial resistance underscores the urgent need for alternative treatments. Ionic liquids (ILs), which are salts composed of cations and anions that remain liquid at or near room temperature, have emerged as promising alternative antimicrobial agents. Their highly tunable nature, achieved by modifying ion combinations, allows for the development of ILs with potent antimicrobial activity, such as choline and geranate (CAGE). However, they can be cytotoxic at concentrations near the therapeutic effective dose, thereby limiting their clinical application. Moreover, recent microbiology advances highlight the need for agents that can effectively target polymicrobial biofilms, which exhibit greater resistance to treatment than their planktonic counterparts. Therefore, this study aimed to optimize IL properties based on the CAGE framework by modifying their ionic composition to enhance their antibiofilm efficacy while improving their biocompatibility. Fatty acids, commonly found in food, cosmetics, and skincare products, were selected as alternative anion donors. Fatty acid-based ILs, particularly choline and oleic acid ([Cho]­[Ole]) and choline and linoleic acid ([Cho]­[Lin]), exhibited strong antimicrobial and antibiofilm efficacy at markedly lower concentrations. In a mouse model, topical application of these ILs significantly reduced subgingival infection. Furthermore, these new ILs demonstrated broader safety margins and caused no tissue irritation even after repeated applications. Therefore, rational ion pairing with fatty acid anions may enhance both ILs’ safety and efficacy, making fatty acid-based ILs promising candidates as next-generation topical agents for periodontal therapy.

## Graphical abstract

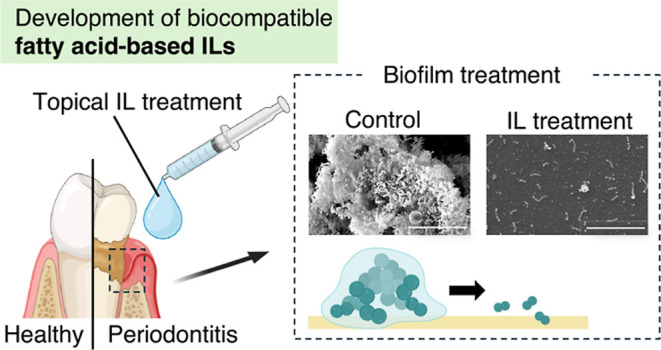

## 1 Introduction

Periodontitis is a highly prevalent, irreversible chronic inflammatory disease that involves the gradual destruction of tooth-supporting structures, ultimately causing tooth loss. Beyond its localized effects on oral function and appearance,^1^ periodontitis has been increasingly recognized for its systemic health implications. It is closely linked to diabetes and has been identified as a risk factor for various systemic diseases, including cardiovascular disease and rheumatoid arthritis.^2,3^ Globally, severe periodontitis affects nearly 19% of adults, accounting for over 1 billion individuals.^4^ Given its substantial health burden and global prevalence, the development of effective therapeutic approaches for periodontitis remains a major public health priority.

Periodontitis begins with the colonization of pathogenic microorganisms in deep periodontal pockets.^5^ Therefore, eliminating the pathogen is fundamental in periodontal therapy. Both systemic and local antibiotic therapies have been widely accepted as adjuncts to mechanical procedures, such as scaling and root planing.^6^ However, the global emergence of antimicrobial resistance (AMR) has become a serious public health concern; thus, the appropriate use of antibiotics and the development of alternative antimicrobials are urgently needed.^7,8^

Ionic liquids (ILs), which are salts composed entirely of cations and anions that remain in a liquid state at or near room temperature, have recently emerged as a promising novel biomaterial and an alternative to conventional antimicrobial agents.^9^ Their key feature is their high tunability achieved through tailored ion pair combinations.^10^ Through their flexible tunability that allows for task-specific optimization at the molecular level,^11^ ILs with significant antimicrobial properties can be developed. In particular, choline and geranate (CAGE) IL outperforms all others.^12^ CAGE can completely neutralize 47 different skin infection pathogens, including drug-resistant pathogens at low concentrations.^13^ Notably, it has also shown strong bactericidal activity against periodontopathic bacteria without promoting AMR.^14,15^

Despite their effectiveness, antimicrobial ILs exhibit toxicity, a major challenge for their widespread medical application.^16^ Cytotoxicity emerges at concentrations effective for antimicrobial use, limiting their clinical potential.^17^ Advances in microbiology also emphasize the need to move beyond treatments targeting planktonic cells and to develop therapeutics that specifically target biofilm.^18,19^ Most of the pathogens exist as polymicrobial biofilms firmly attached to the tooth surface rather than as free-floating planktonic cells.^20^ Pathogens residing in biofilms are embedded in a self-produced extracellular polymeric substance (EPS), which shields them from host immune responses and chemical drug exposure.^21^ Additionally, subgingival biofilms serve as reservoirs of virulence factors and toxins that elicit complex and sustained host immune responses.^22^ Therefore, next-generation IL-based periodontal therapeutics must be developed to achieve both antimicrobial and antibiofilm efficacy, while also being biocompatible for potential clinical translation.

This study aimed to enhance CAGE functionality by modifying its ionic composition to meet the above-mentioned critical criteria. The antimicrobial action is primarily attributed to its geranate anion;^23^ thus, we explored alternative fatty acids as anion donors. Fatty acids are essential components of cell membranes and common dietary lipids. Some fatty acids exhibit antimicrobial activity against oral bacteria.^24^ By utilizing the various properties of fatty acids, we were able to synthesize five new fatty acid-based ILs. Their antimicrobial, antibiofilm, and biocompatibility profiles were then evaluated and compared with those of CAGE. Among them, the most potent candidates were identified and selected for further evaluation in animal models.

## 2 Results and Discussion

### 2.1 Synthesis and Characterization of the ILs

Five fatty acids, namely, azelaic acid, octanoic acid, lauric acid, oleic acid, and linoleic acid, were selected as alternative anion donors to geranate. These fatty acids were chosen primarily based on their biocompatibility and physiological relevance. We focused on compounds that are naturally present in biological systems (e.g., octanoic acid, linoleic acid) or widely used as safe components in pharmaceutical and cosmetic formulations. In addition, the selected fatty acids represent a range of carbon chain lengths and hydrophobicity, enabling a systematic evaluation of how the anionic structure modulates the physicochemical and potential therapeutic properties of the synthesized ILs. Among the synthesized fatty acid-based ILs, choline-azelaic acid ([Cho]­[Aze]) IL and choline-octanoic acid ([Cho]­[Oct]) IL formed low-viscosity liquids, whereas choline-lauric acid ([Cho]­[Lau]), choline-oleic acid ([Cho]­[Ole]) and choline-linoleic acid ([Cho]­[Lin]) formed high-viscosity liquids (Figure 1).

**Figure 1:**
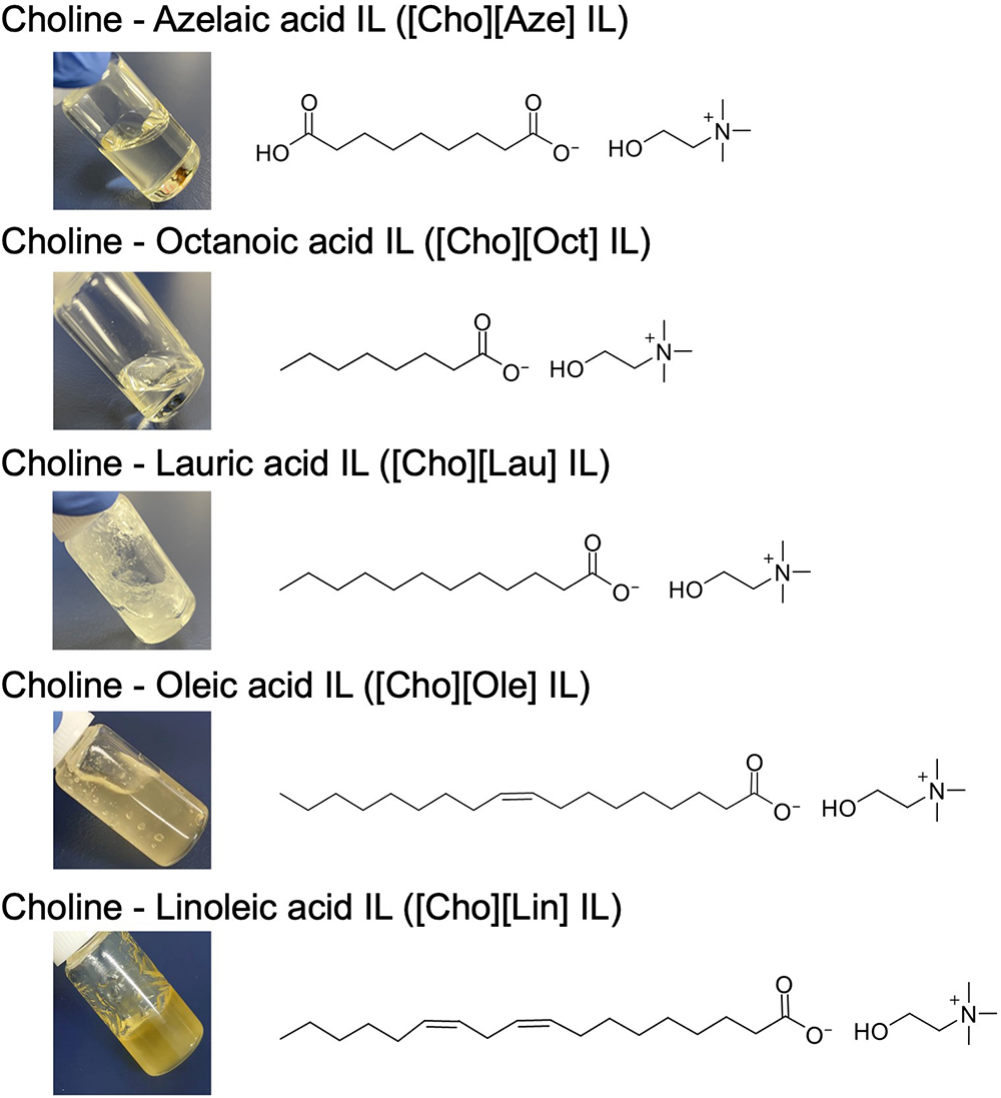
Photos and structures of the new fatty acid-based ILs.

Regarding the purity and structures of the ILs, the ^1^H nuclear magnetic resonance (NMR) spectra showed that the carboxylic proton disappeared from the free fatty acids and that both the choline and fatty acid signals demonstrated characteristic chemical shift changes, consistent with IL formation. The expected choline signals (N^+^(CH_3_)_3_ at −3.2 ppm, –CH_2_OH at 3.4–3.5 ppm) and methylene/methyl peaks of the fatty acids were clearly observed, with spectral patterns reflecting the chain length of the fatty acids and the degree of unsaturation (Figures S1–S5). Meanwhile, the Fourier transform infrared (FT-IR) spectra of the free choline bicarbonate and each fatty acid exhibited characteristic peaks attributable to the –OH and C=O groups at approximately 3000–3300 and 1700 cm^–1^, respectively. Given that the 80% choline bicarbonate reagent contains water, the –OH peak of choline appeared as a broad band. In the ILs, the C=O peak around 1700 cm^–1^ decreased, and the N–O stretching peak around 1550 cm^–1^ slightly changed. These peak shifts indicate intermolecular interactions between choline and the fatty acids, attributed to IL formation (Figure 2).

**Figure 2:**
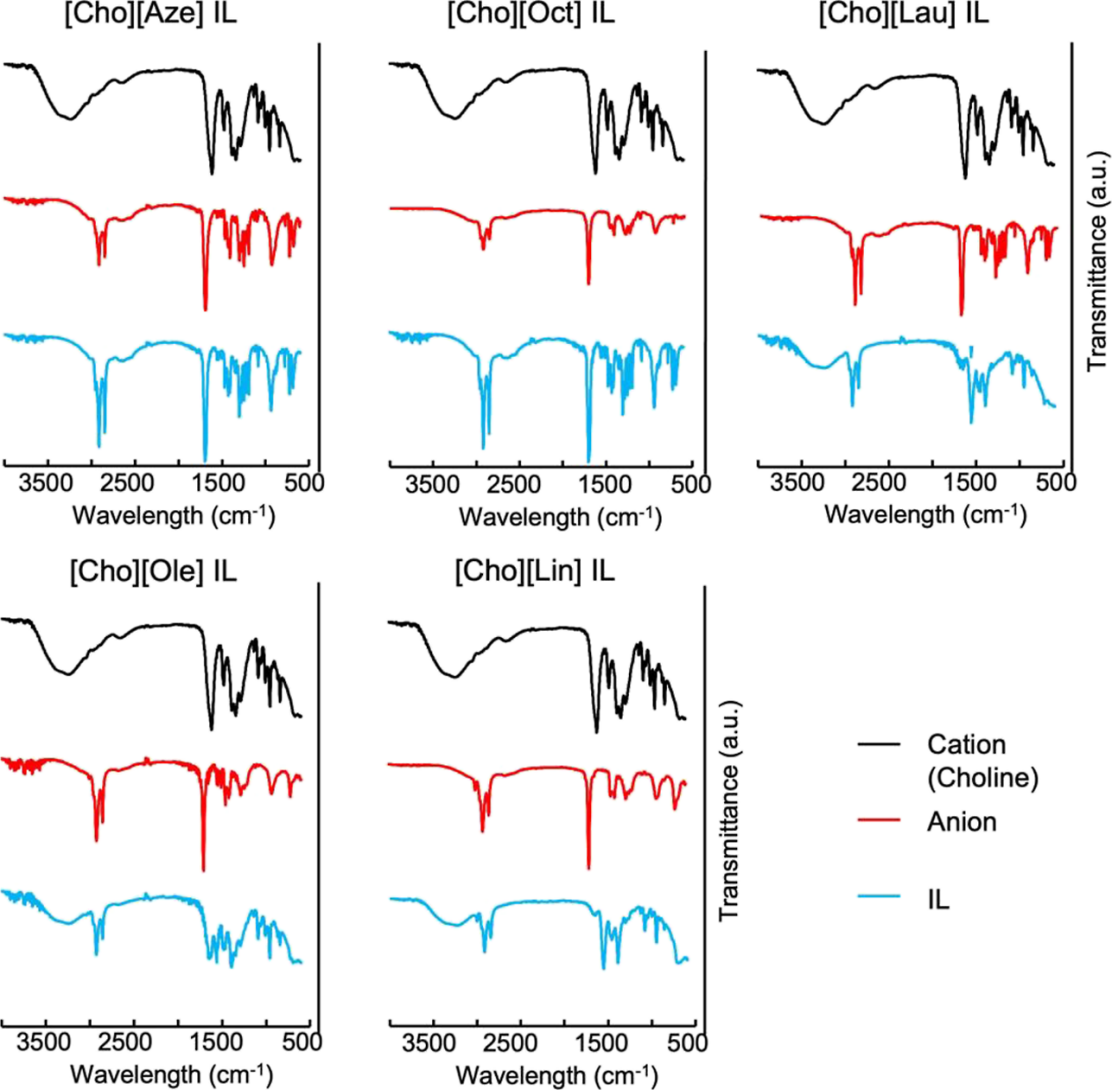
FT-IR spectra of the fatty acid-based ILs.

### 2.2 Screening of the Fundamental Properties of the Fatty Acid-Based ILs

In examining fatty acid-based ILs’ general antimicrobial activity, we measured the minimum inhibitory concentration (MIC) against *Porphyromonas gingivalis*, a major periodontopathic bacterium (Table 1). The MIC of CAGE was also measured and found to be consistent with a previous report.^15^ All new ILs demonstrated antimicrobial efficacy equal to or greater than that of CAGE. In particular, [Cho]­[Lau], [Cho]­[Ole], and [Cho]­[Lin] ILs exhibited significantly stronger antimicrobial activity, with their MICs being 30–125 times lower than that of CAGE.

**Table 1:** Screening of the Fundamental Properties of the Fatty Acid-Based ILs

IL	MIC (μg/μL)	MCC (μg/μL)	MCC/MIC
[Cho][Aze] IL	1.25	40	32
[Cho][Oct] IL	1.25	40	32
CAGE	1.25	5	4
[Cho][Lau] IL	0.04	2.5	64
[Cho][Ole] IL	0.02	2.5	125
[Cho][Lin] IL	0.01	1.25	125

The antimicrobial efficacy and in vitro biocompatibility of ILs were evaluated by determining the MIC against *P. gingivalis* and the MCC against human gingival epithelial cells (*n* = 3). All replicates produced identical end point values; therefore, MIC and MCC are presented as single representative values. The safety margin (MCC/MIC) represents the range between the antibacterial and cytotoxic concentrations. MIC, minimum inhibitory concentration, MCC, minimum cytotoxic concentration.

Subsequently, cytotoxicity toward gingival epithelial cells was assessed. Table 1 lists the minimum cytotoxic concentration (MCC) of each IL. ILs with stronger antimicrobial activity tended to induce cytotoxicity at lower concentrations, consistent with the fact that ILs’ antimicrobial action primarily results from the disruption of the lipid bilayer, which can also interact with host cell membranes.^28^

However, the safety margin, which refers to the range between antibacterial and cytotoxic concentrations (MCC/MIC), notably differed among the ILs. CAGE exhibited a relatively narrow safety margin, with cytotoxicity occurring at only 4 times the MIC (MCC/MIC = 4). Conversely, the new ILs demonstrated markedly broader safety margins (MCC/MIC = 32–125), particularly [Cho]­[Ole] and [Cho]­[Lin] ILs. Of note, a wider safety margin indicates a greater potential for safe and effective use.

Overall, the new ILs exhibited potent antimicrobial activities along with improved biocompatibility compared with CAGE. These properties were further investigated in the following experiments.

### 2.3 Antimicrobial and Antibiofilm Activities of the Fatty Acid-Based ILs

The ILs’ antimicrobial potential was further confirmed by assessing their minimum bactericidal concentration (MBC) against *P. gingivalis* (Figure S6). All of the new ILs presented bactericidal efficacy. Consistent with the MIC results, [Cho]­[Ole] and [Cho]­[Lin] ILs exhibited particularly stronger bactericidal activity at lower concentrations than CAGE.

The antibiofilm activity of the new ILs was examined by treating established biofilms with a range of concentrations for 10 min, followed by cell viability evaluation using the live/dead assay (Figure 3A). All of the new ILs showed significant reductions in the ratio of live (green) to dead (red) cells, proving their bactericidal effect against biofilm-embedded bacteria. Conversely, chlorhexidine gluconate (CHG), a disinfectant commonly used in mouthwash products, showed no significant bactericidal efficacy at its clinical concentration. Moreover, [Cho]­[Ole] and [Cho]­[Lin] ILs’ minimum concentrations required for biofilm neutralization (minimum Live/Dead) were remarkably lower than those of the others (Figure 3B).

**Figure 3:**
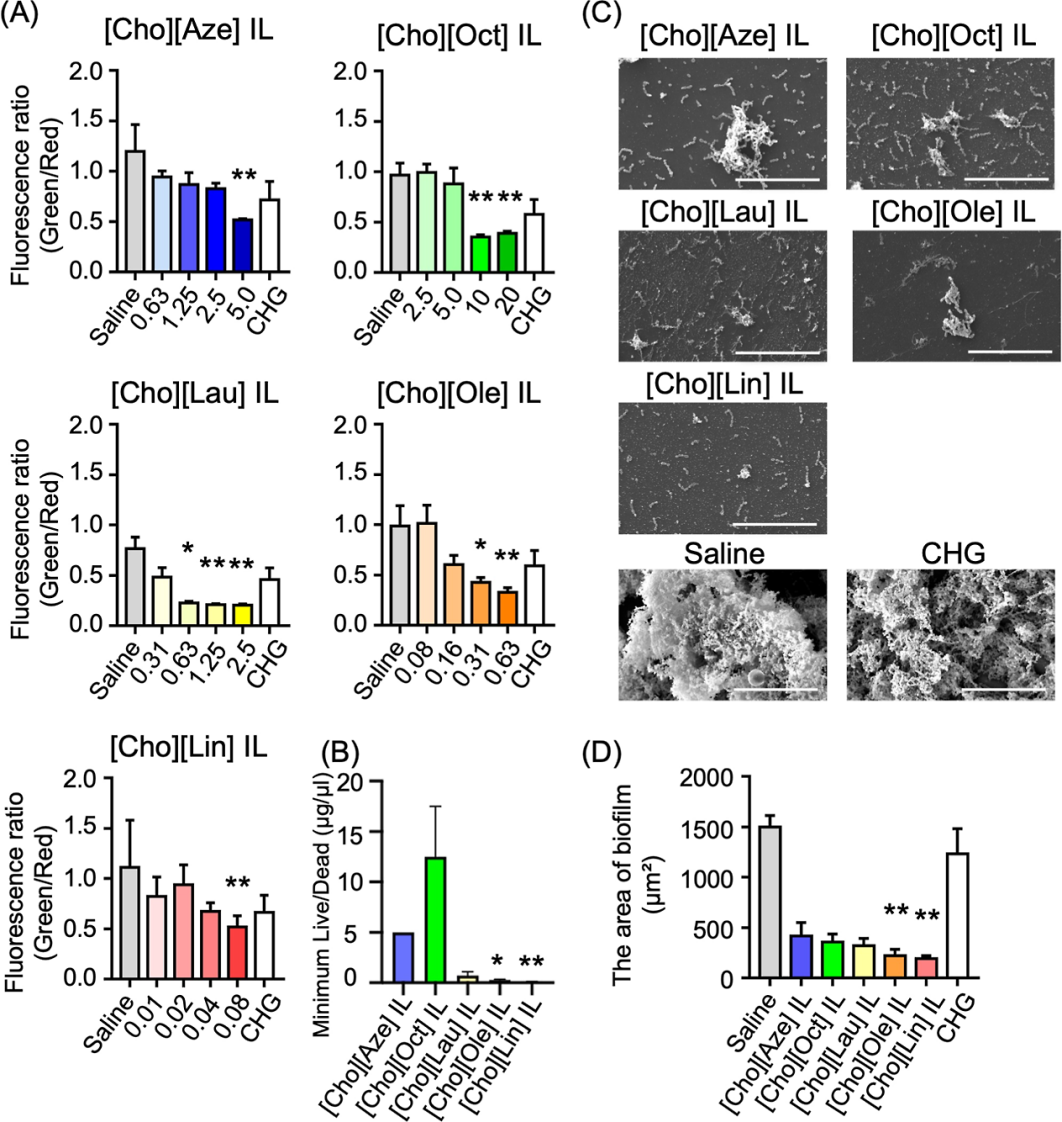
Antibiofilm efficacy of the new ILs in the *P. gingivalis* biofilm model. (A) Cell viability within the biofilm after IL exposure. ILs were tested at concentrations within 0.01–20 μg/μL for 10 min. The ratio of live cells (green: 480/500 nm excitation/emission) to dead cells (red: 490/635 nm) was calculated (*n* = 4). Significant difference (Kruskal–Wallis test followed by Dunn’s multiple comparison test): vs saline, *P < 0.05, **P < 0.01. Chlorhexidine gluconate (CHG) at 0.01% served as the control. (B) Comparison of the minimum concentrations of ILs required for biofilm neutralization (minimum live/dead) (*n* = 3). Significant difference (Kruskal–Wallis test followed by Dunn’s multiple comparison test): *P < 0.05, **P < 0.01 (vs [Cho]­[Oct]). (C) Representative SEM images of the biofilm after IL treatment at the minimum live/dead. Scale bars: 20 μm. (D) Biofilm area on SEM images taken at random locations, measured using ImageJ (*n* = 4). Significant difference (Kruskal–Wallis test followed by Dunn’s multiple comparison test): vs saline, **P < 0.01.

In addition, the scanning electron microscopy (SEM) images of IL-treated biofilm samples revealed that the biofilm structure was disrupted after IL treatment at the minimum live/dead concentrations (Table S1), whereas in the control and CHG-treated samples, it remained intact (Figure 3C,D). A quantitative analysis of the biofilm area supported this observation, showing significant reductions, particularly after treatment with [Cho]­[Ole] and [Cho]­[Lin] ILs. No reduction in antibiofilm efficacy was observed even after approximately one year of storage, indicating that the IL formulations maintained their activity over time.

Taken together, the fatty acid-based ILs demonstrated to be promising antibiofilm agents that effectively neutralize biofilm pathogens and disrupt biofilm structures. Given that current clinical agents face significant limitations in biofilm treatment because of the inherent drug tolerance of biofilms,^29^ these ILs represent a promising alternative capable of overcoming these barriers.

This drug tolerance primarily stems from two key factors: poor drug penetration into the biofilm matrix and the phenotypic transformation of biofilm bacteria. The EPS functions as a physical barrier, preventing the diffusion of clinically used antibiotics and other antimicrobial agents to pathogens located deep within the biofilm.^30,31^ For example, although CHG has substantial antimicrobial effects against oral pathogens, it penetrates slowly, merely reaching the outer layers of the biofilm.^32^ Consistent with this finding, CHG did not show significant efficacy against biofilms in our study. Additionally, bacteria within biofilms adopt a distinct phenotype that increases their AMR by up to 1000 times compared with their planktonic counterparts,^30^ making biofilm-related infections particularly difficult to treat with conventional therapeutics.

Conversely, the fatty acid-based ILs effectively overcome the limitations of conventional periodontal agents by inducing biofilm structural disruption and bacterial inactivation through a physical disruption mechanism. Although the molecular mechanisms underlying the antibiofilm activity of the newly synthesized fatty acid-based ILs have not been directly confirmed, ion–matrix interactions are expected to play a key role. Based on previous reports, the proposed mechanism likely begins with electrostatic interactions between the positively charged choline cation and the negatively charged biofilm surface.^10^ Subsequently, the ionic nature of these compounds enables them to penetrate dense by inserting their hydrophobic moieties into the hydrophobic domains of the EPS,^10,33^ resulting in the loosening or partial disruption of the matrix structure. This disruption facilitates deeper drug penetration and enhances biofilm clearance, as confirmed by our assays. Moreover, once in contact with biofilm-embedded bacteria, ILs function through a nonspecific physical mechanism, disrupting membrane integrity and inducing pore formation.^34^ This mechanism imposes minimal selective pressure, making it effective even against phenotypically resistant bacteria within biofilms. Direct evaluation of ionic interactions and molecular dynamics within the biofilm matrix remains to be elucidated and represents a limitation of this study. Future work will focus on clarifying these molecular processes.

### 2.4 Enhanced Efficacy of ILs over Corresponding Fatty Acids

Given that some of the fatty acids themselves exhibit antimicrobial activity, their efficacy was evaluated using the same methods and compared with that of the corresponding ILs to assess the benefit of IL conversion. The fatty acids had significantly higher MBCs than their respective ILs, indicating lower antimicrobial potency (Figure S7). The difference was even more notable in the biofilm assays (Figure 4). The minimum Live/Dead values of the fatty acids were 65–250 times higher than those of the corresponding ILs, demonstrating ILs’ superior efficacy against biofilms. Furthermore, azelaic acid and lauric acid exhibited inhomogeneous dispersion in the medium, making stable results difficult to obtain.

**Figure 4:**
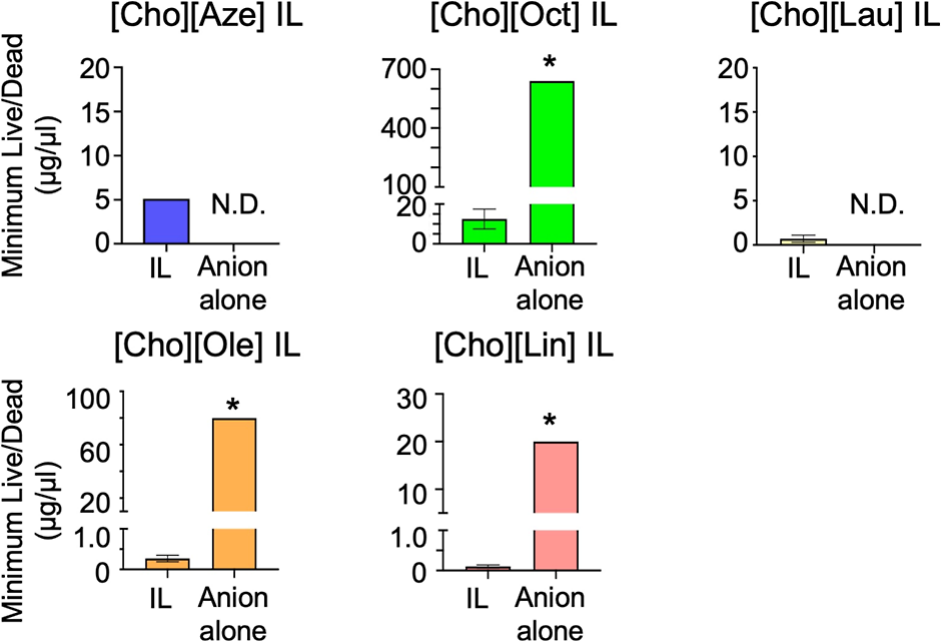
Comparison of the antibiofilm efficacy between the ILs and their corresponding anions. The minimum concentrations required for biofilm neutralization (minimum live/dead) for each IL and its corresponding anion are shown (*n* = 4). Azelaic acid and lauric acid showed poor dispersion in the medium, hindering the acquisition of consistent results. Significant difference (Mann–Whitney *U* test): *P < 0.05. N.D.: No data.

Overall, IL conversion may enhance both antimicrobial and antibiofilm activities compared with the corresponding fatty acids.

### 2.5 Repeated-Dose Toxicity Study In Vivo

To assess the in vivo biocompatibility of the new ILs, we applied the ILs (5 μL, at the minimum live/dead concentration) once daily for 1 week to the healthy gingival tissues around the molar teeth of the mouse upper jaw (Figure 5A). Body weight change and water and food intake were monitored throughout the experimental period to assess the systemic impact of local IL application (Figure 5B–D). The body weight in the [Cho]­[Lin] IL group slightly decreased only on day 3, while no significant differences in body weight and water and food intake were observed at any other time point compared with the saline-treated control.

**Figure 5:**
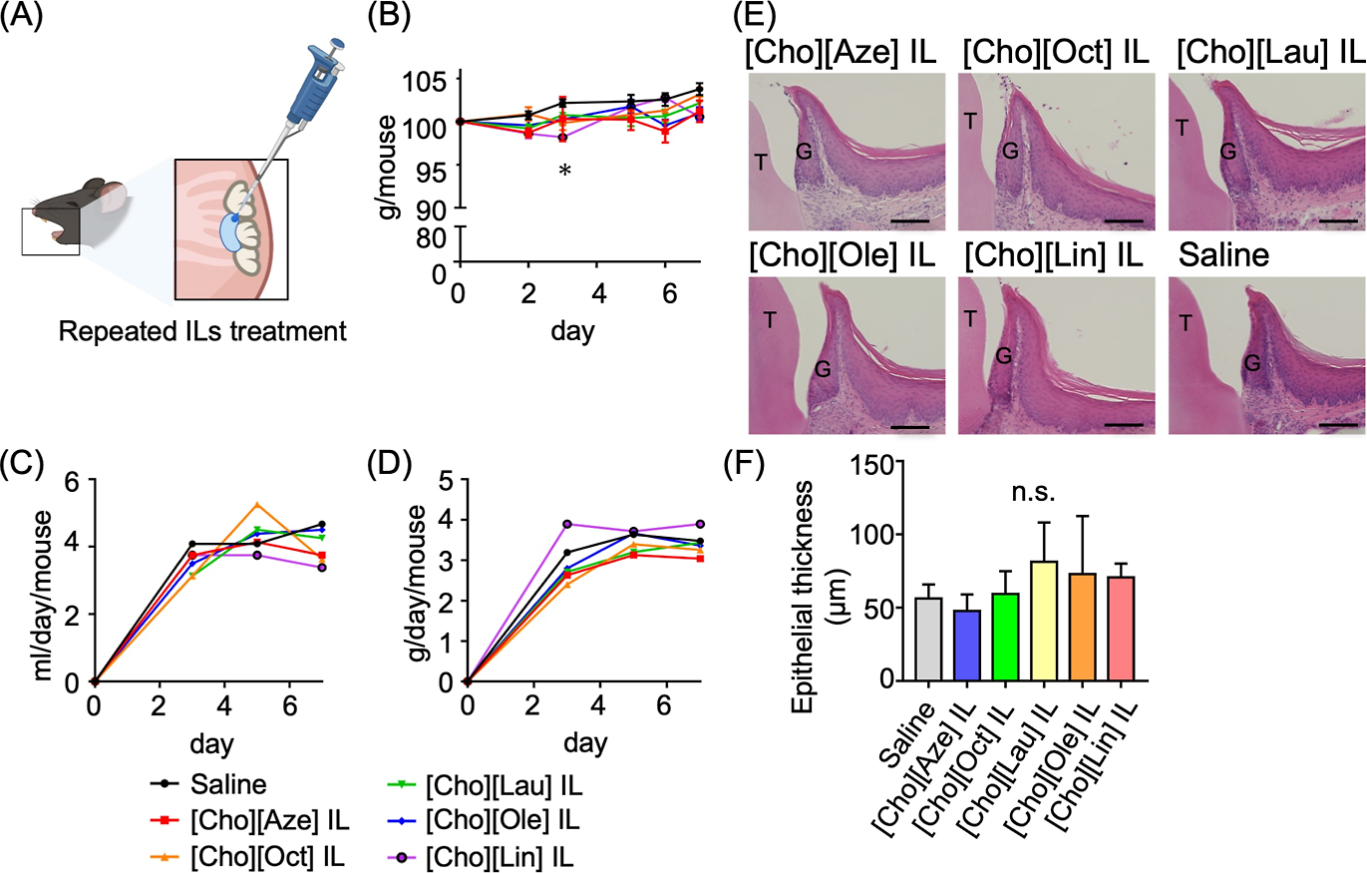
Biocompatibility test in vivo. (A) Schematic of the biocompatibility test. Healthy gingival tissues around the upper molar teeth were repeatedly treated with ILs (5 μL at the minimum live/dead concentrations, daily for 1 week). (B) Body weight change (*n* = 4). Significant difference (Kruskal–Wallis test followed by Dunn’s multiple comparison test): vs saline, *P < 0.05. (C) Average daily water intake, and (D) average daily food intake. (E) Representative H-E-stained periodontium at the test sites after repeated treatment. Scale bars: 100 μm. (F) Thickness measurement of the gingival epithelia on the H–E-stained sections (*n* = 4). Significant difference (Kruskal–Wallis test followed by Dunn’s multiple comparison test): vs saline, *P < 0.05. T: Tooth, G: Gingiva, n.s.: not significant.

At the end of the treatment period, the upper jaws were harvested for histological examination by H–E staining to assess local tissue responses (Figure 5E,F). None of the IL-treated samples showed inflammatory cell infiltration, angiogenesis, or other tissue irritation signs. Moreover, the epithelial thickness did not significantly differ between the IL-treated and saline-treated tissues, indicating no epithelial hyperplasia (Figure 5F). These histological parameters were used as representative criteria for evaluating local irritation or tissue toxicity, confirming the good biocompatibility of the ILs.

Therefore, repeated topical application of the ILs at antibiofilm concentrations did not induce significant tissue damage. This finding is particularly important, given the high risk of recolonization of periodontal pathogens in the periodontal pockets.^35^ Consistent oral care and potentially repeated antimicrobial interventions are necessary to prevent recolonization.^33^ The absence of detectable tissue injury even after daily IL application highlights the potential of ILs as safe agents for the long-term management of periodontal infections.

### 2.6 Infection Model Study In Vivo

The most promising candidates, exhibiting potent anti-infective activity and good biocompatibility, were identified according to the antimicrobial and biocompatibility results and selected for further evaluation in an animal infection model. Specifically, [Cho]­[Lau], [Cho]­[Ole], and [Cho]­[Lin] ILs were chosen. To mimic pathogenic infection in the subgingival area, we inserted bacteria into the gingival sulcus of the upper front teeth, followed by topical application of the ILs (Figure 6A). Bacterial DNA was collected before and after the treatment and analyzed by quantitative polymerase chain reaction (qPCR). While topical treatment with CHG did not yield significant improvement compared with the control, treatment with ILs, particularly [Cho]­[Ole] and [Cho]­[Lin], significantly decreased the *P. gingivalis* 16S rRNA gene copy number (Figure 6B,C).

**Figure 6:**
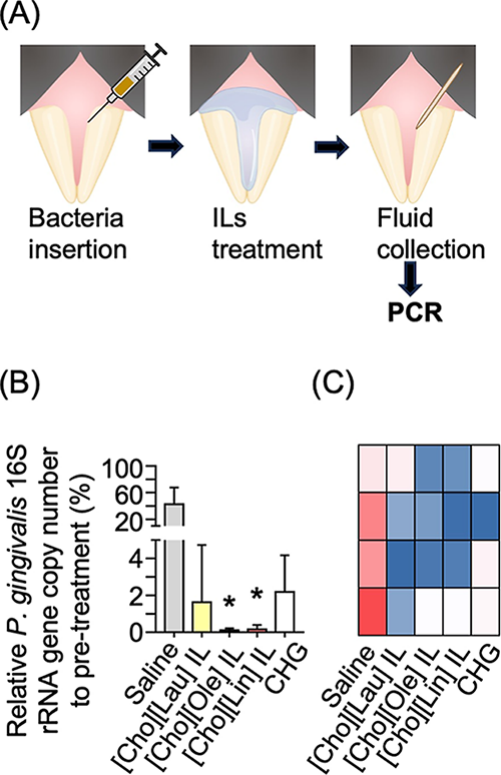
Infection model study in vivo. (A) Schematic of the infection model, treatment, and analysis. The infected subgingival regions of the upper front teeth in mice were treated with the three most effective ILs identified in vitro, saline, or 0.01% CHG for 15 min. We collected fluid samples from the sites before and after treatment. The gene copies of *P. gingivalis* 16S rRNA in the fluid samples were analyzed using qPCR (*n* = 4). (B) Relative *P. gingivalis* 16S rRNA gene copy number to pretreatment. Significant difference (Kruskal–Wallis test followed by Dunn’s multiple comparison test): vs saline, *P < 0.05. (C) Heatmap for gene copies of the *P. gingivalis* 16S rRNA in the fluid samples. Columns indicate treatment groups, and rows indicate individual mice (*n* = 4). Red and blue denote higher and lower gene copy numbers, respectively.

Therefore, the topical application of ILs effectively reduced pathogenic infection in the subgingival area. However, of note, periodontopathic bacteria rarely colonize the mouse oral cavity, and a well-established mouse model that fully replicates the complex periodontal biofilm observed in humans is currently lacking.^36^ Hence, further research is required to evaluate the in vivo antibiofilm efficacy of ILs in more clinically relevant models. In addition, future studies employing established periodontitis models will be needed to assess the therapeutic and healing effects of ILs for clinical translation, as well as to further evaluate their in situ stability under more physiologically relevant conditions.

### 2.7 Influence of the Anion Structure on the IL Antimicrobial Potency

To explore the basis of the different antimicrobial and antibiofilm activities among the ILs, we analyzed the key chemical characteristics of the anions (Table 2), including molecular weight, carbon chain length, water solubility, and p*K*_a_, in relation to the MIC or minimum live/dead values of the corresponding ILs. The MIC values negatively correlated with the molecular weight and carbon chain length but positively correlated with the water solubility; meanwhile, no clear relationship was observed with p*K*_a_ (Figure 7A). The minimum Live/Dead values also negatively correlated with the molecular weight and chain length but showed no association with the water solubility or p*K*_a_ (Figure 7B).

**Table 2:** Key Chemical Features of the Anions

anion	molecular weight	carbon number	water solubility	p*K*_a_
azelaic acid	188.2209	9	2.28	4.15
octanoic acid	144.2114	8	0.91	5.19
geranate	168.2328	10	1.22	5.26
lauric acid	200.3178	12	0.01	4.95
oleic acid	282.4614	18	0.00012	4.99
linoleic acid	280.4455	18	0.00015	4.99

**Figure 7:**
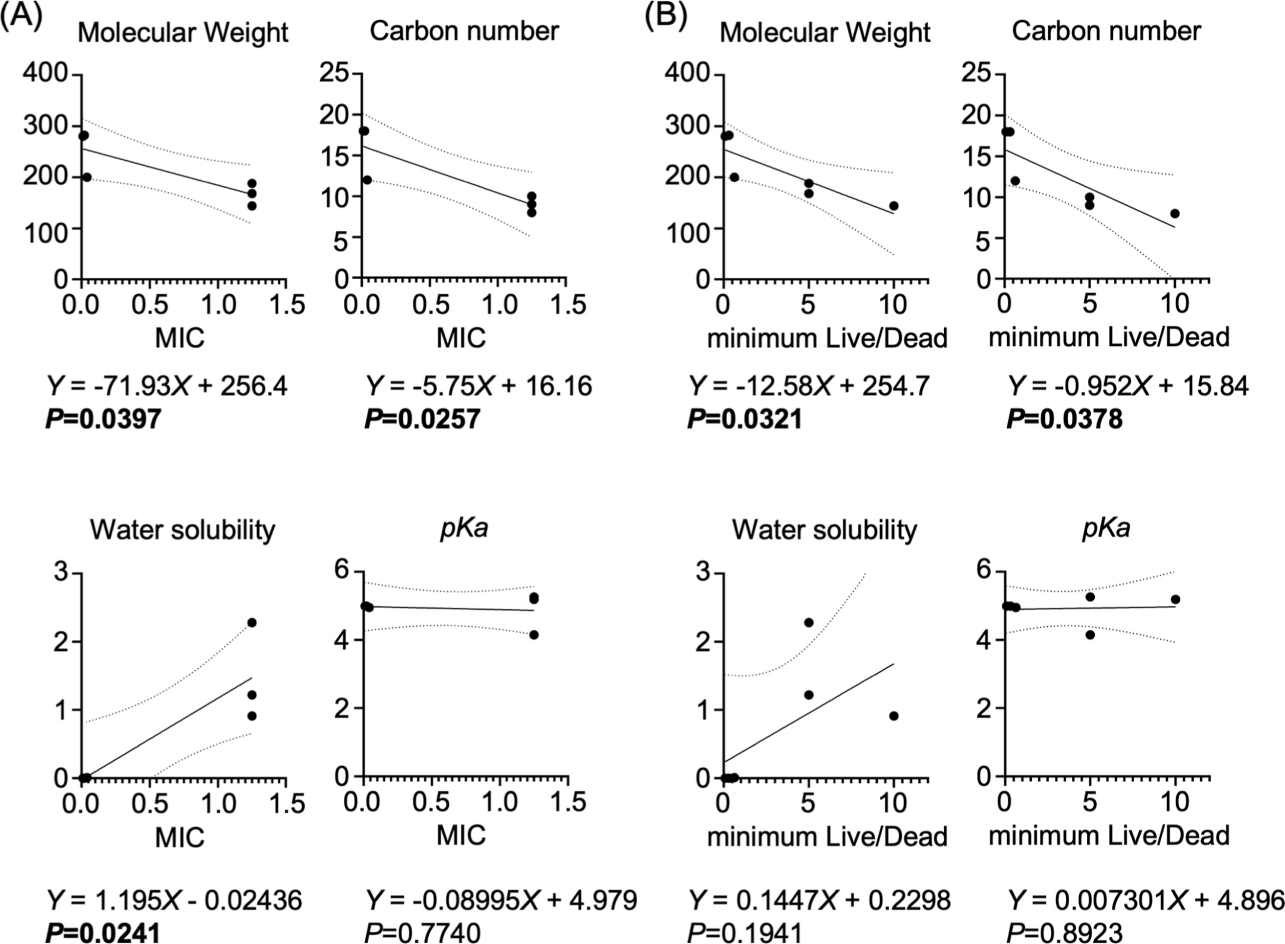
Correlation between the anion chemical properties and the antimicrobial/antibiofilm efficacy. (A) Relationship between the MIC values and the anion chemical features analyzed by simple linear regression. (B) Relationship between the minimum concentration required for biofilm neutralization (minimum live/dead) and the anion chemical features analyzed by simple linear regression. Statistically significant *P* < 0.05. MIC, minimum inhibitory concentration.

While the influence of the cation structure on the IL activity has been extensively documented,^37^ reports on the impact of the anion structure remain limited.^36^ Our findings demonstrate that the anion structure also contributes to IL potency. In previous studies on IL cations, a longer aliphatic alkyl chain enhanced antimicrobial activity by facilitating interactions with and disruption of bacterial membranes.^38,39^ Consistent with this concept, our results show that anions with higher carbon numbers also increase antimicrobial effects.

Although hydrophobicity is crucial in the antimicrobial activity by facilitating lipid bilayer disruption, it did not exhibit a clear correlation with antibiofilm activity. This finding likely reflects the complex composition of the biofilm EPS matrix, which contains both hydrophilic and hydrophobic components. Additionally, given the minimal variation in the p*K*_a_ values of fatty acid anions, no relationship with antimicrobial activity was found.

These results highlight that the antimicrobial and antibiofilm activities of the newly synthesized ILs are determined by the structural and physicochemical properties of the anionic component, suggesting that careful selection of anions with longer carbon chains and favorable physicochemical properties may further enhance the IL efficacy.

## 3 Conclusions

In this study, we optimized IL properties based on the CAGE framework by modifying their ionic combinations to develop new-generation periodontal therapeutics (Figure 8). Fatty acids showed to be promising anion donors, given that fatty acid-based ILs exhibited strong antimicrobial and antibiofilm activities, as well as good biocompatibility, a wide safety margin, and no evidence of tissue irritation after repeated application. The [Cho]­[Ole] and [Cho]­[Lin] ILs showed particularly high potential.

**Figure 8:**
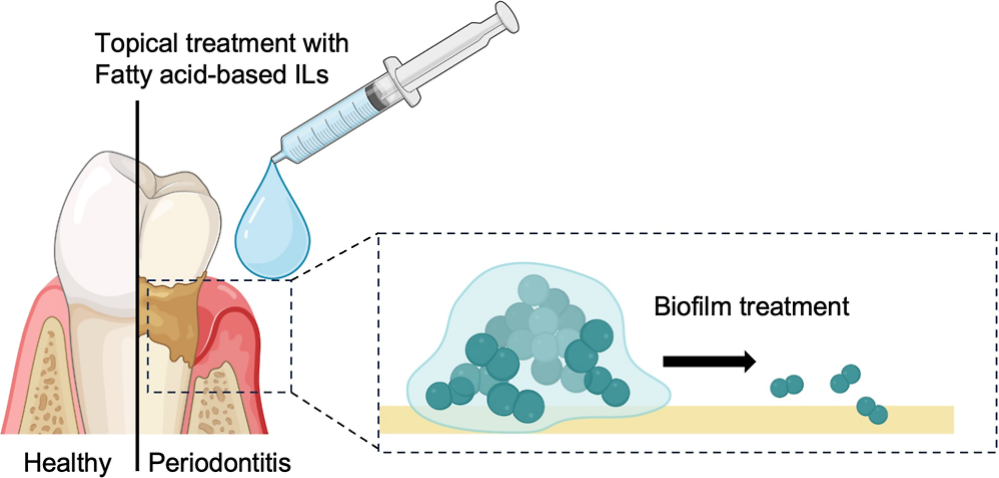
Schematic illustration of topical periodontal therapy using biocompatible fatty acid-based ILs.

Nevertheless, several limitations should be acknowledged. First, the detailed molecular mechanisms underlying the antibiofilm effects of fatty acid-based ILs remain to be fully elucidated. Second, while the in vivo efficacy of these ILs was confirmed, validation in more clinically relevant models is still required. Additionally, further refinement of ion combinations may lead to the discovery of even more effective IL-based therapeutics. Despite these limitations, the present findings provide new insights into the development of IL-based therapeutics, underscoring both the strength and novelty of this approach in addressing biofilm-associated periodontal disease.

## 4 Materials and Methods

### 4.1 Materials

The anaerobic jar and AnaeroPack were purchased from Mitsubishi Gas Chemical Co. Inc. (Tokyo, Japan). Dulbecco’s Modified Eagle Medium (DMEM), 4-(2-hydroxyethyl)-1-piperazineethanesulfonic acid (HEPES), Live/Dead BacLight Bacterial Viability Kit, and SYBR Green qPCR Master Mix were purchased from Thermo Fisher Scientific (MA, USA). We also purchased penicillin–streptomycin (PN-st), CHG, paraformaldehyde-phosphate1 buffer (PFA), and hematoxylin and eosin (H-E) from FUJIFILM Wako Pure Chemical Corporation (Osaka, Japan), and thiazolyl blue tetrazolium bromide (MTT), geranate, choline bicarbonate, oleic acid, and OSTEOSOFT from Sigma-Aldrich (MO, USA). Azelaic acid, octanoic acid, and linoleic acid were purchased from Tokyo Chemical Industry Corporation (Tokyo, Japan).

We procured other materials from their respective manufacturers as follows: Gifu anaerobic medium (GAM) from Nissui (Tokyo, Japan), fetal bovine serum (FBS) from NICHIREI BIOSCIENCES Inc. (Tokyo, Japan), lauric acid from Nacalai Tesque Inc. (Kyoto, Japan), Cryomatrix from Epredia Holdings Ltd. (NH, USA), and C57BL/6NJ mice from The Jackson Laboratory Japan, Inc. (Kanagawa, Japan).

### 4.2 Synthesis of Fatty Acid-Based ILs

We used choline bicarbonate as the cation, and geranate and the other fatty acids (azelaic acid, octanoic acid, lauric acid, oleic acid, and linoleic acid) as the anions. Choline bicarbonate was slowly added to the fatty acids at a molar ratio of 1:1, except for oleic acid and geranate, which required 2:1 and 1:2 ratios to form ILs, respectively. We then stirred the mixture at 40 °C for 30 min. Residual water was removed by rotary evaporation (ELYLA N-1300, Tokyo Rikakikai, Tokyo, Japan) at 60 °C for 2 h (40 °C for oleic acid and linoleic acid), followed by drying in a vacuum oven at 60 °C for 48 h (at room temperature for oleic acid and linoleic acid). The synthesized [Cho]­[Ole] and [Cho]­[Lin] ILs were kept under refrigerated conditions (4 °C), whereas the other fatty acid-based ILs were stored at room temperature. No visible changes such as discoloration, viscosity alteration, or crystallization were observed during storage for up to one year. We employed FT-IR Spectroscopy (Shimadzu, Kyoto, Japan) and NMR (JEOL 400YH, Tokyo, Japan) to determine the purities and structures of the synthesized compounds. ^1^H NMR spectra were recorded in methanol or chloroform as solvents.

### 4.3 Bacterial Culture and Biofilm Formation

*P. gingivalis* ATCC33277 was cultured in modified GAM broth under anaerobic conditions using an AnaeroPack at 37 °C for 48 h, until reaching a density of 1 × 10^9^ colony forming units (cfu)/mL. To form biofilms, we added 100 μL of *P. gingivalis* suspension (1 × 10^9^ cfu/mL) and 100 μL of GAM broth to a 96-well plate, which was subsequently incubated statically at 37 °C under anaerobic conditions for 3 days until the biofilm matured.

### 4.4 Measurement of the MIC and MBC

MIC was measured using a broth microdilution method.^25^ The bacterial suspension was diluted to 2 × 10^7^ cfu/mL and then mixed 1:1 with serial 2-fold dilutions of ILs (0.01–20 μg/μL). We transferred 200 μL of the mixture to a sterile 96-well plate, yielding final IL concentrations of 0.005–10 μg/μL. The control wells contained bacteria in 200 μL of GAM without ILs. After incubation at 37 °C for 24 h under anaerobic conditions, bacterial growth was assessed by measuring the absorbance at 600 nm using a microplate reader (SpectraMax ABS Plus, Molecular Devices, CA, USA). The lowest concentration that inhibited bacterial growth served as the MIC. To measure MBC, we first plated 10 μL of the culture from each well onto blood agar, followed by anaerobic incubation at 37 °C for 5–7 days. The lowest concentration at which no bacterial growth was observed on the plate served as the MBC. All MIC and MBC determinations were performed in triplicate (*n* = 3).

### 4.5 Cell Culture and MTT Assay

The human gingival epithelial cell line Ca9–22 was cultured as previously described.^26^ Briefly, supplemented with 10% FBS and 1% PN-st, the cells were maintained in DMEM containing HEPES. They were seeded in 96-well plates at a density of 1 × 10^4^ cells/well, cultured for 24 h, and subsequently treated with 100 μL of ILs (0.078–160 μg/μL) for 1 h. Thereafter, we replaced the medium with MTT solution and incubated the cells for 2 h. The absorbance was then measured at 570 nm using a SpectraMax ABS Plus to assess IL cytotoxicity (*n* = 3). All cultures were maintained at 37 °C in a 5% CO_2_ atmosphere.^27^

The minimum cytotoxic concentration (MCC) was defined as the lowest IL concentration that induced a significant reduction in cell viability in the MMT assay. The safety margin was calculated as the ratio of the MCC to the MIC (MCC/MIC) indicating how many times higher than the antibacterial effective concentration the compound can be applied without causing cytotoxicity.

### 4.6 Bacterial Cell Viability Test

Bacterial cell viability within the biofilm was evaluated using the Live/Dead BacLight Bacterial Viability Kit (*n* = 4). We treated the biofilm with either 200 μL of saline, a serial dilution of ILs (0.01–20 μg/μL), or 0.01% (=0.1 μg/μL) CHG for 10 min, followed by washing and staining with a working solution of SYTO9 and propidium iodide (PI) at a 1:1 ratio for 15 min in the dark. Additionally, we measured fluorescence at an excitation wavelength of 475 nm, with emission detected at 530 nm for SYTO9 (live cells, green fluorescence) and 630 nm for PI (dead cells, red fluorescence). To determine cell viability, we calculated the ratio of green (live cells) to red (dead cells) fluorescence.

### 4.7 SEM Imaging

Biofilms formed on the round glass coverslips. After being treated with ILs (at the minimum live/dead concentration, as determined by the live/dead assay), saline, or 0.01% CHG for 10 min, the samples were washed with PBS, fixed with 2.5% glutaraldehyde for 15 min, and dehydrated in ethanol solutions (50%–100%) serially for 15 min each. Before SEM analysis, the samples were air-dried using a critical point dryer (HCP-2; Hitachi, Tokyo, Japan) and sputter-coated with a Pt/Pd alloy using an ion sputter coater (E1030; Hitachi, Tokyo, Japan). SEM (SU3700N; Hitachi, Tokyo, Japan) at a beam voltage of 10 kV was utilized for imaging. The SEM images were further processed using the Fiji/ImageJ software, with the area of the cell aggregates quantified (*n* = 4).

### 4.8 Animals

Male C57BL/6NJ mice (7–10 weeks old) were acclimatized under specific pathogen-free conditions and provided with regular chow and sterile water throughout the experiment. All experiments conformed to the study protocol approved by the Institutional Animal Care and Use Committee at Niigata University (Permit no.: SA01326), the Regulations and Guidelines on Scientific and Ethical Care and Use of Laboratory Animals of the Science Council of Japan, and the ARRIVE guidelines.

### 4.9 Biocompatibility Test In Vivo

Under isoflurane inhalation anesthesia, animals’ healthy gingival tissues around the upper molar teeth were repeatedly treated with 5 μL of ILs (at the minimum Live/Dead concentration) once daily for 1 week (*n* = 4). Body weight change and water/food intake were monitored throughout the experimental period. At the end of the treatment period, we harvested the upper jaw and prepared tissue sections as described below.

### 4.10 Histological Analysis

The harvested upper jaw was fixed in 4% PFA for 24 h, decalcified in OSTEOSOFT for 48 h, embedded in Cryomatrix, and sectioned thinly (7–8 μm thick) in the sagittal direction along the teeth’s long axis. The sections were stained with H-E and imaged using a BZ-X710 fluorescence microscope (KEYENCE, Osaka, Japan). We further processed each image using Fiji/ImageJ and measured the epithelial thickness (*n* = 4).

### 4.11 Infection Model Study In Vivo

We established the in vivo infection model according to a previously reported method with minor modifications.^33^ Briefly, using a microsyringe, we inserted *P. gingivalis* (1 × 10^9^ cfu/mL, 10 μL) into the gingival sulcus region of the upper front teeth (*n* = 4). The inoculum size was chosen to approximate the bacterial load typically detected in periodontal pockets of patients with periodontitis.^40^ Next, the three most effective ILs identified in vitro (at the minimum live/dead concentration, 5 μL), saline, or 0.01% CHG was applied on to the gingival tissues for 15 min to treat the infection site. Bacterial DNA was collected by sterile paper points #35 before and after the treatment. The paper points were placed in 200 μL of PBS, and the bacterial DNA was extracted by vibration for 30 min. The isolated DNA suspension was used for qPCR, which was performed using the QuantStudio 1 Real-Time PCR Instrument (Thermo Fisher Scientific) with Fast SYBR Green Master Mix. The custom-designed oligonucleotide sequences targeting the *P. gingivalis* 16S rRNA gene were as follows: forward, AGGCAGCTTGCCATACTGCG; reverse, ACTGTTAGCAACTACCGATGT. To estimate the bacterial genome count at the infection site, we converted the Ct values obtained from the qPCR into gene copy numbers.

### 4.12 Statistical Analysis

Unless otherwise noted, data in graphs are presented as mean ± standard deviation, with GraphPad Prism 8.3 (GraphPad Software, Inc., San Diego, CA, USA) used for statistical analyses. Comparison between two groups was conducted using the Mann–Whitney *U* test. For multiple-group comparisons, we employed the Kruskal–Wallis test followed by Dunn’s multiple comparison test. Simple linear regression was used to analyze the correlations of the MIC values and the minimum live/dead values with the chemical features of the fatty acids. Moreover, *P < 0.05 and **P < 0.01 indicated statistical significance.

## Supplementary Material



## Notes

^1^H NMR spectra of Choline–Azelaic acid IL ([Cho]­[Aze] IL) (Figure S1), ^1^H NMR spectra of Choline–Octanoic acid IL ([Cho]­[Oct] IL) (Figure S2), ^1^H NMR spectra of Choline–Lauric acid IL ([Cho]­[Lau] IL) (Figure S3), ^1^H NMR spectra of Choline–Oleic acid IL ([Cho]­[Ole] IL) (Figure S4), ^1^H NMR spectra of Choline–Linoleic acid IL ([Cho]­[Lin] IL) (Figure S5), Minimum bactericidal concentrations (MBCs) of the ILs against *P. gingivalis* (Figure S6), Comparison of MBC between the ILs and their corresponding anions (Figure S7), and The Minimum Live/Dead concentrations of the IL (Table S1) (PDF)
