# Supplementary material for: Development of Biocompatible Fatty Acid-Based Ionic Liquids for the Effective Topical Treatment of Periodontitis
Source: ACS Omega. 2025 Dec 29;11(1):1495–504. doi: 10.1021/acsomega.5c09114 (PMC12809864; doi:10.1021/acsomega.5c09114)
Supplement: Supplementary file 1 [file ao5c09114_si_001.pdf]

## Supplementary Materials

# Development of Biocompatible Fatty Acid–based Ionic Liquids for the Effective Topical Treatment of Periodontitis

Mayuko Yanagawa [a], Mayuka Nakajima [a] *, Mayumi Ikeda-Imafuku [b], Tatsuya Fukuta [b], Kotone Yoshimura [b], Chunyang Yan [a], Lorena Zegarra-Caceres [a], Honoka Takikawa [a], Truong T. Thien [a], Ruka Koizumi [a], and Koichi Tabeta [a] *

[a] Division of Periodontology, Faculty of Dentistry & Graduate School of Medical and Dental Sciences, Niigata University, Niigata 951-8514, Japan
[b] Department of Physical Pharmaceutics, School of Pharmaceutical Sciences, Wakayama Medical University, 25-1 Shichiban-cho, Wakayama 640-8156, Japan

This file includes the following:

Figure S1. $^{1}$H NMR spectra of choline-azelaic acid ionic liquid ([Cho][Aze] IL)

Figure S2. $^{1}$H NMR spectra of choline-octanoic acid ionic liquid ([Cho][Oct] IL)

Figure S3. $^{1}$H NMR spectra of choline-lauric acid ionic liquid ([Cho][Lau] IL)

Figure S4. $^{1}$H NMR spectra of choline-oleic acid ionic liquid ([Cho][Ole] IL)

Figure S5. $^{1}$H NMR spectra of choline-linoleic acid ionic liquid ([Cho][Lin] IL)

Figure S6. Minimum bactericidal concentrations (MBCs) of the ILs against *Porphyromonas gingivalis*.

Figure S7. Comparison of MBCs between the ILs and their corresponding anions.

Table S1. The Minimum Live/Dead concentrations of the ILs.

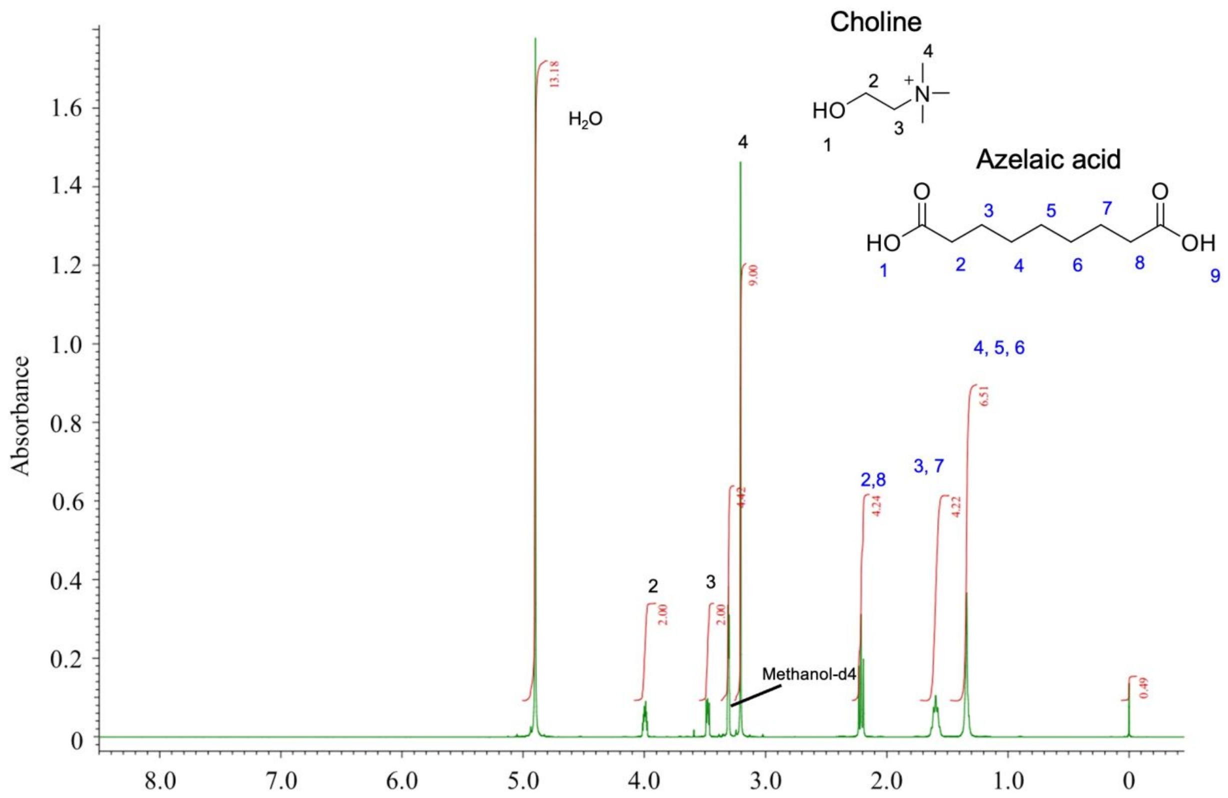


**Figure S1.** $^{1}H$ NMR spectra of choline-azelaic acid ionic liquid ([Cho][Aze] IL)

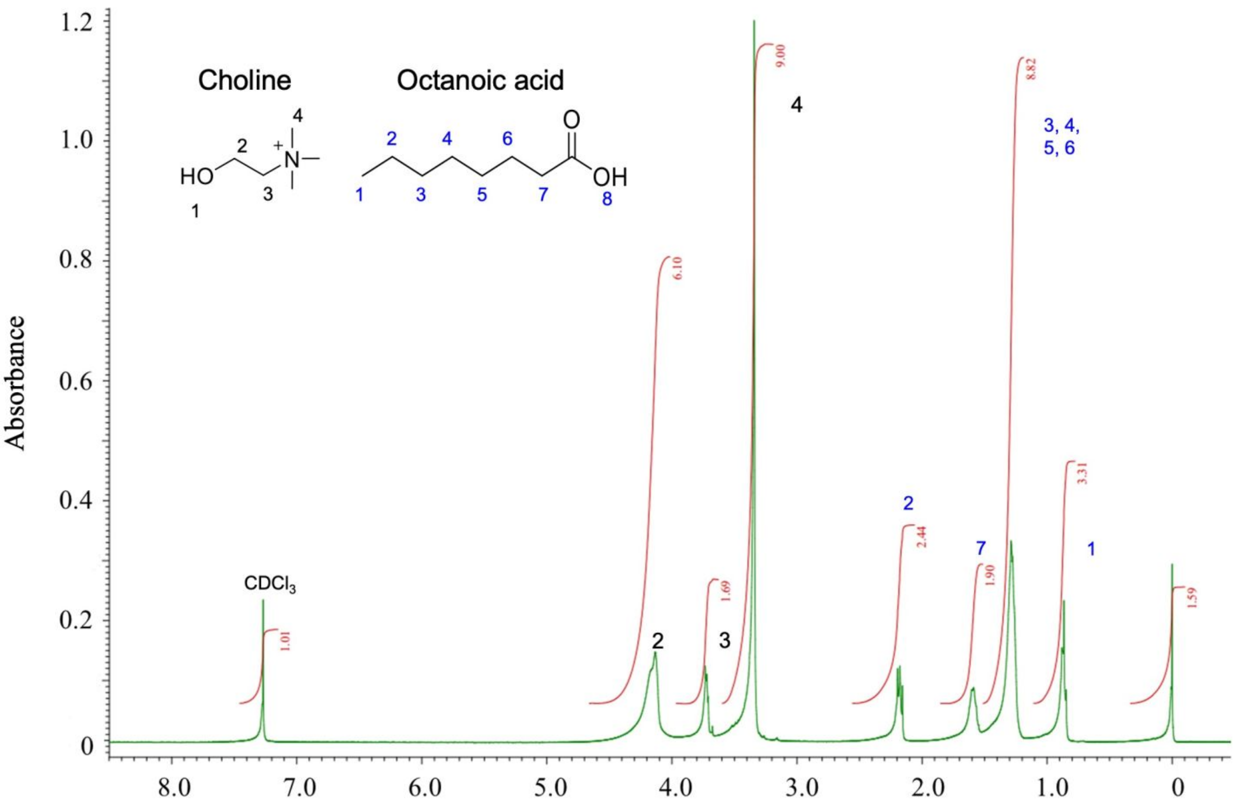


**Figure S2.** $^{1}$H NMR spectra of choline-octanoic acid ionic liquid ([Cho][Oct] IL)

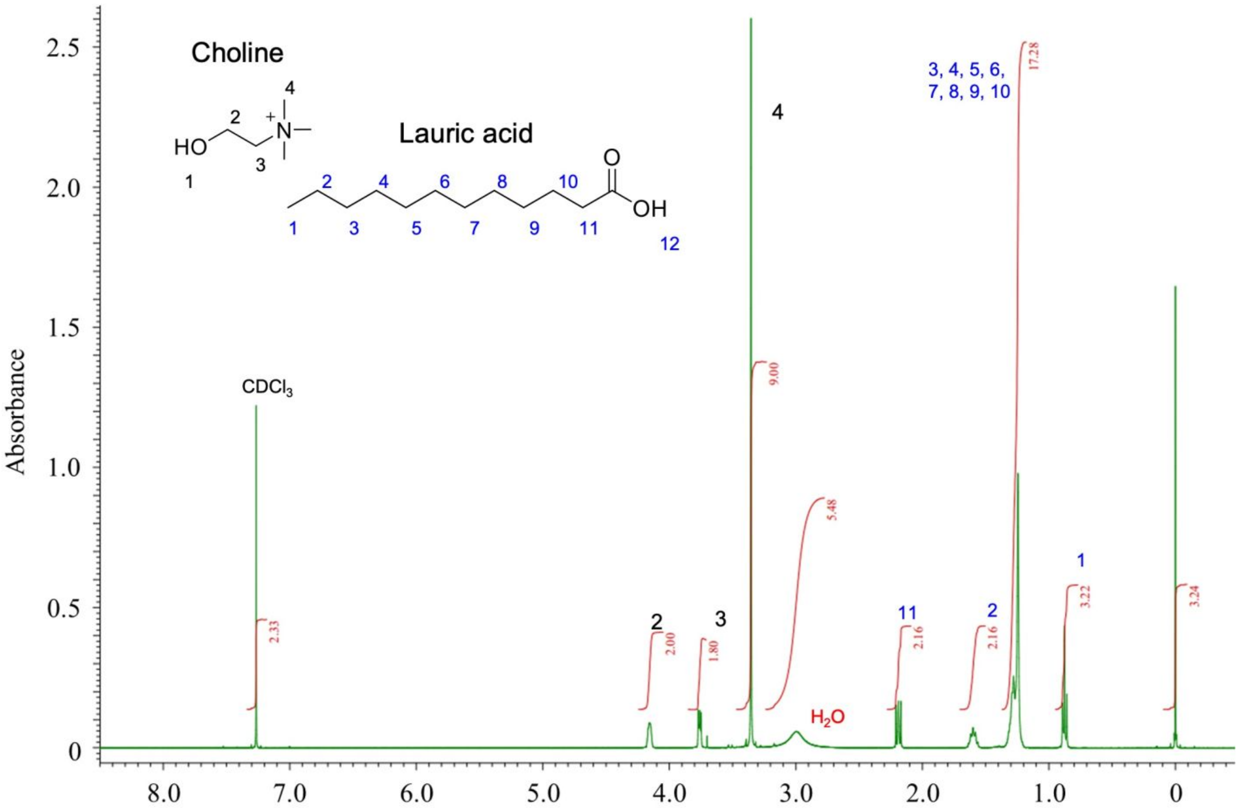


**Figure S3.** $^1$H NMR spectra of choline-lauric acid ionic liquid ([Cho][Lau] IL)

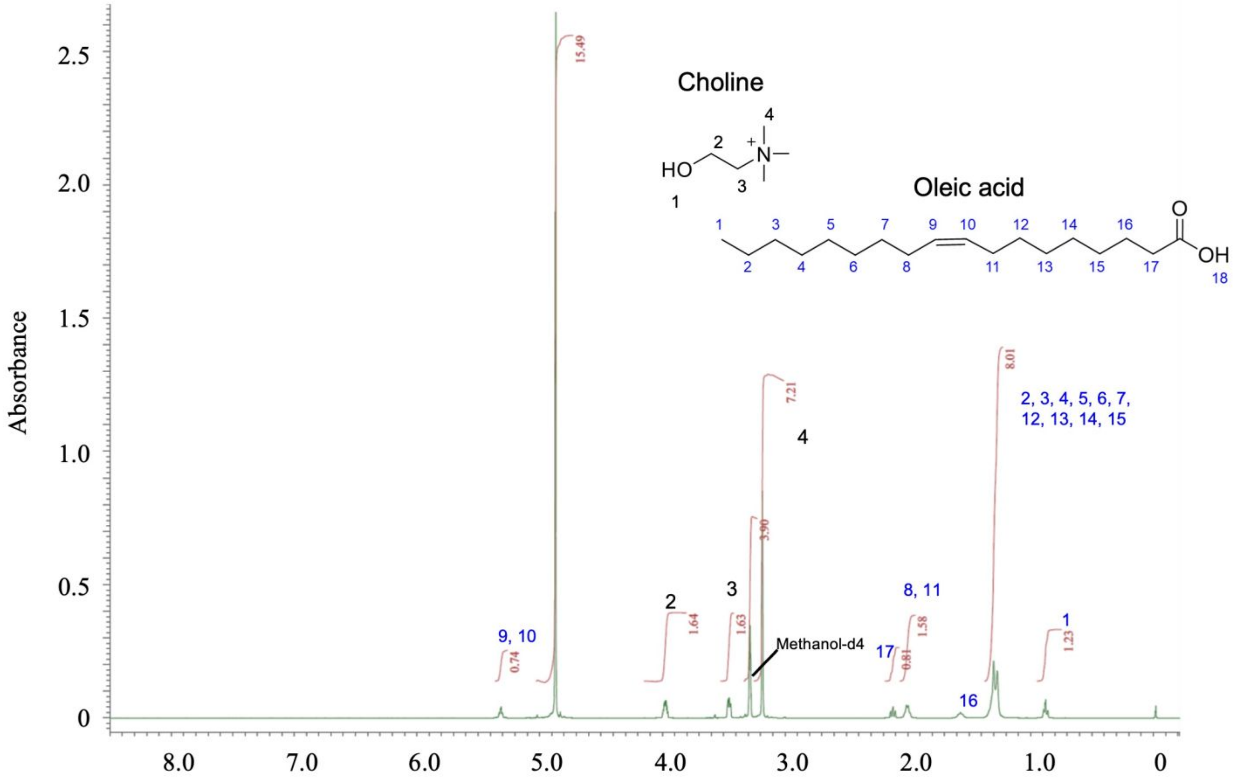


**Figure S4.** $^{1}H$ NMR spectra of choline-oleic acid ionic liquid ([Cho][Ole] IL)

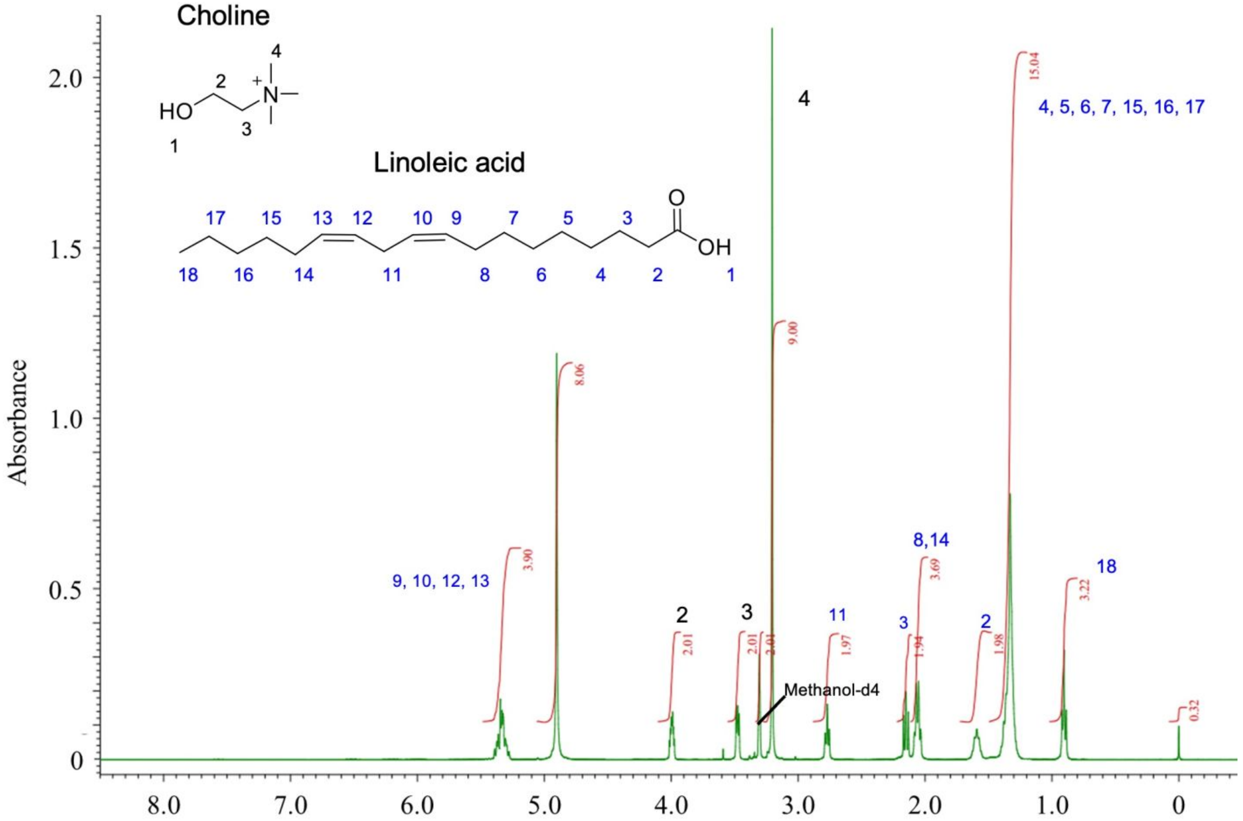


**Figure S5**. $^1$H NMR spectra of choline-linoleic acid ionic liquid ([Cho][Lin] IL)

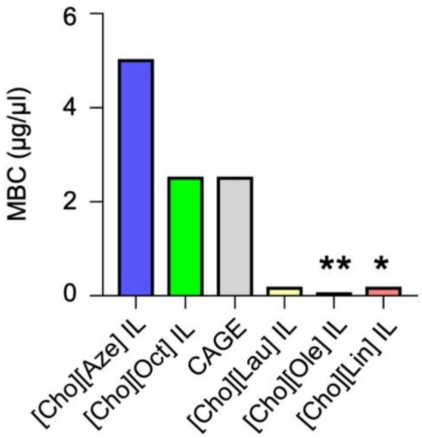


**Figure S6.** Minimum bactericidal concentrations (MBCs) of the ILs against *Porphyromonas gingivalis*. All new ionic liquids (ILs) demonstrated bactericidal efficacy, with [Cho][Ole] and [Cho][Lin] ILs exhibiting particularly strong effects at lower concentrations. $n = 3$. Significant difference (Kruskal–Wallis test followed by Dunn's multiple comparison test), $*P < 0.05$, $**P < 0.01$.

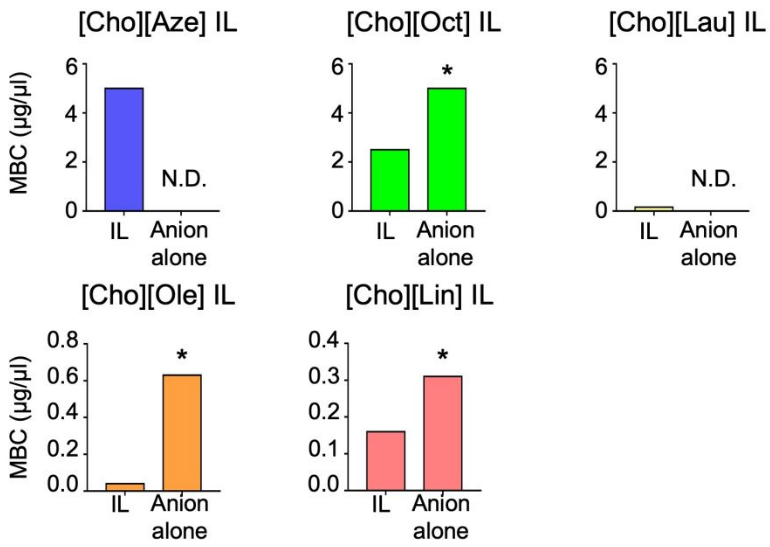


**Figure S7.** Comparison of MBCs between the ILs and their corresponding anions. MBCs against *P. gingivalis* for each IL and its anion alone are shown ($n = 4$). Azelaic acid and lauric acid showed poor dispersion in the medium, making the results inconsistent. Significant difference (Mann–Whitney *U* test): $*P < 0.05$. N.D.: No data

Table S1. The Minimum Live/Dead concentrations of the ILs.

| IL | Minimum Live/Dead concentration (μg/μl) |
|---|---|
| [Cho][Aze] IL | 5 |
| [Cho][Oct] IL | 10 |
| [Cho][Lau] IL | 0.63 |
| [Cho][Ole] IL | 0.31 |
| [Cho][Lin] IL | 0.08 |

List of the Minimum Live/Dead concentrations used for subsequent analyses.
